# Factor structure and psychometric properties of the Hungarian version of the Mentalized Affectivity Scale (MAS): A cross-sectional study

**DOI:** 10.1371/journal.pone.0329785

**Published:** 2025-08-06

**Authors:** Veronika Bóné, Éva Kalla, Attila Pilinszki

**Affiliations:** 1 Institute of Mental Health, Semmelweis University, Budapest, Hungary; 2 Faculty of Health and Public Services, Pázmány Péter Catholic University, Budapest, Hungary; Aalborg University, DENMARK

## Abstract

**Background:**

There is an increasing need among researchers to explore how emotions are regulated and processed. Mentalized affectivity is a complex cognitive and affective ability to understand thoughts, feelings and intentions of oneself and others.

**Aims:**

This study proposes a psychometric validation of the Hungarian version of the Mentalized Affectivity Scale (MAS), and measures the three main domains of mentalized emotions: emotion recognition, processing and expression.

**Materials & methods:**

Participants (N = 316) were recruited using the snowball sampling method via various mailing lists and social networking sites from August 2023 to November 2023. Structural Equation Modeling was used to validate the original factor structure and Principal Component was used for subsequent exploration. We used Spearman’s correlation to measure convergent validity and the Kruskal-Wallis test for correlations with demographics.

**Results:**

Initial confirmatory and exploratory factor analysis on the 60 items of Greenberg did not show satisfactory outcomes. Subsequently, we checked the 35-item version of the scale: after deleting one item, and using the original three-factor structure, we found that the scale showed great internal consistency and sufficient convergent validity to other measured constructs.

**Discussion & conclusion:**

The Hungarian 34-item Mentalized Affectivity Scale and its three subscales are suitable for measuring mentalized affectivity.

## Introduction

### Emotion regulation and mentalization

Emotion regulation is a fundamental skill for healthy mental functioning [1–5], involving biologically based explicit and implicit processes that involve monitoring, evaluation and modulation of emotions [6]. It is also one of the fastest developing areas of psychology because of its role in personal well-being and social relationships [7]. Currently, there is an extensive theoretical framework for understanding regulatory and processing processes [8], although the theoretical frameworks do not form a unified picture [9]. Greenberg et al. [10] highlight that recent theories of regulation [11] emphasize the ability to change strategies in a flexible way, but they also point to the developmental psychopathology perspective as an important direction [12], which argues that early childhood life experiences have a lasting impact on emotion regulation. Related to this is the mentalized affectivity (MA) concept of Jurist [13], which further enriches the range of theories by integrating the high level of mentalization skills into the process of emotion regulation, arguing that regulation relies heavily on mentalization. The mentalization theoretical framework is now a widely known and applied concept in both research and clinical practice. Its conceptualization is described by theorists as a complex cognitive and affective ability to understand thoughts, feelings and intentions of oneself and others, a social competence [14–16]. Liotti et al. [17] summarize the development of the mentalization theory framework and highlight that some aspects of it share similarities with other known constructs. To improve its measurability, Fonagy and Target [18] operationalized it as a reflective function. As mentalization matures, it leads to the experience of understanding social relationships, establishing a foundation for lived trust, which is essential for social learning [19,20]. Rinaldi et al. [21] summarize the role of mentalization in emotion regulation, arguing that through mentalization, emotion regulation is achieved through the continuous reappraisal of affect, whereby we better understand the complexity of affect. Schwartzer et al. [22] conducted correlational studies in a non-clinical sample to clarify the relationship between mentalization and emotion regulation. Their results suggest that self-focused reflective capacity is a prerequisite for adaptive emotion regulation, predicting the quality of emotion regulation. Their results are also important because they confirm the basic concept of mentalization theory, according to which throughout development. the capacity of the caregiver for mentalization is crucial in the development of emotion regulation skills and in the organization of the self [16]. Its advanced functioning in adulthood facilitates the emergence of mentalized affect [23].

Regarding the environmental factors influencing emotion regulation, Rinaldi et al. [21] summarize that the normative of regulation differs between Eastern and Western cultures, and that this difference is also reflected in mentalization, although little research is known in this area. In a study related to this topic, Jurist et al. [24] argue for the importance of identifying cultural differences in favor of the concept of mentalized affectivity, which combines emotion regulation, mentalization and autobiographical memory. Aviah-Navel, Rothshild -Yakar & Kumar [25] found cross-cultural differences when reviewing concepts that overlap with the dimensions of mentalization (Theory of Mind (ToM), empathy and perspective-taking, alexithymia, mindfulness), which are presumably due to differences in language, parenting practices and values.

### Mentalized affectivity

Fonagy et al. [16] described mentalized affectivity as a sophisticated form of affect regulation. According to Jurist [9] mentalised affectivity helps us to recognise meaningful events and situations through reflection and understanding of experienced emotions. Seeing what is happening in a wider context and anticipating expected reactions. The three main elements of regulation in the identification, processing and expression of affect include openness and curiosity to understand and rework it [26]. Jurist states that the identification of affect is achieved through mentalizing it in the context of the person’s particular circumstances and memories. It also implies the recognition of the origin of the emotion, not just the naming of the emotion. Emotion processing refers to the tolerance and modulation of emotions, with variations in duration and intensity, as well as the careful differentiation of complex emotions. While the expression of emotions can be outward and inward adaptive. It is explicitly about regulating one’s own emotions, not understanding the emotions of others [9]. Jurist [9] highlights that mentalized affectivity involves our emotional experiences as lived and interpreted at a deeper level in the context of our life history and environment. Greenberg et al. [10] summarize that, in this, it is related to autobiographical memory, through which early experiences are influenced by the current reactions of the person. Jurist [9] points out that mentalized affectivity helps us to recognize meaningful events and situations, to better understand what is happening, and to anticipate expected reactions. In this context, Fonagy et al. highlight that mentalized affectivity can facilitate communication, supporting the ability to adapt to different situations [16], and thus promote resilience [26]. Levante et al. [27] investigated well-being and, in relation to this, mentalized affectivity during the quarantine period due to the Covid-19 virus and found that mentalizing had a beneficial effect on the well-being of teachers. That is, as theorists have argued, mentalized affectivity also contributes to well-being [24,28].

### Measuring mentalized affectivity

A number of different approaches have been used to measure affectivity, including the DERS (Difficulties in Emotion Regulation Scale [29]), ERQ (Emotion Regulation Questionnaire [30]), EROS (Emotion Regulation of Others and Self [31]), ASQ (Affective Style Questionnaire [32]), ERSQ (Emotion-Regulation Skills Questionnaire [33]), RF (Reflective Functioning Questionnaire [34]) AII (Affect Integration Inventory [35]) ACI & ACS (Affect Consciousness Interview; Affect Consciousness Scales [36]), RFS (Reflecting Functioning Scale [18]), PANAS (Positive and Negative Affect Scale [37]), FREE (Flexible Regulation of Emotional Expression [38]), CERG (Cognitive Emotion Regulation Questionnaire [39]), and the MEQ (Mentalizing Emotions Questionnaire [40]).

Greenberg et al. [28] reviewed several of these instruments in search of an appropriate measure of mentalized affectivity and concluded that each approaches mentalizing affectivity from its own perspective, but lacks the threefold structure of identifying, processing and expressing that follows from MA theory [13]. In the next step, four experts who had participated in a pre-training session developed a 76-item scale that covers each of the three main dimensions. Their goal was to prepare a thorough and detailed questionnaire covering all elements of mentalization affectivity that had been identified so far, so they divided the three main elements into seven subcategories: identifying basic (1) and complex (2) affects, modulating (3) and refining (4) affects, outward (5) and inward (6) expressing and (7) prior expreriences. Greenberg et al. note that although the scale might be psychometrically suitable for detailed measurement of mentalization affectivity, one of its main limitations is its length, which may cause potential fatigue in future research, so some items have been deleted. After primary analyses, redundant or poorly worded items were then removed, leaving still 60 items. The Identifying subscale consists of 24 items, Processing 23, and Expressing 13. A total of 15 items are reverse coded [9]. The analyses of Greenberg show that the internal consistency of the scale is high, the threefold structure of the theoretical model is justified, and the subscales are independent of each other.

One drawback of the Mentalized Affectivity Scale is the length of time it takes to complete. It is common research experience in any field of the empirical data collection, that there has been a decrease in the willingness to complete and a shift toward scale shortening [41,42]. Shortening has also been shown to be necessary and reliable for MAS [10], which has been used successfully in situations where mentalized affectivity is part of a pool of multiple measures. The BMAS is composed of 12 items, shortened based on previous analysis [28] using the following criteria: each subscale should contain 4 items each, have at least one reversal between them, not be too highly correlated with each other, and have a high factor loading value in the original factor structure. Overall, the B-MAS also has good internal consistency and is a robust measure of mental affectivity.

The scale (in its shorter or longer form) has been successfully applied to different age groups, such as adolescents [43–45], the elderly [46], different professions, such as students, teachers [27,47], in samples of social workers [48], in attitudinal studies, such as research on discrimination against black Americans [49], in various psychological disorders [50–53], alcoholism [54], narcissism [55], in research on relationships [56], and in child-rearing [57], but also in other areas such as musical training [58], but also in research on covid [27,48], and even in the context of epistemic trust or vaccine beliefs [59].

According to the Mentalized Affectivity Lab, the scale has so far been translated into 16 languages [60]. The Persian-validated version available in international scientific databases contains 60 items [45], the Korean adaptation (K-MAS [61]), has 54 items, the Italian longer version [21] retained 35 items from the original (excluding items that showed low or high factor loadings on several factors in the Italian sample). Additional translations were made into Japanese, Taiwanese, Mandarin, German, Spanish, Turkish, French, Lithuanian, Swedish, and Bulgarian.

The abbreviated scale has also been adapted to other languages, with the Italian version [17,50] confirming the factor structure and robustness of the original abbreviation. However, in the analysis of the Persian version, one of the four items of the identifying factor was excluded, resulting in a scale with 11 items [62].

The factor analysis of the scale has been confirmed by several studies using the Iranian validated version [45,63]; however, some studies of the K-MAS factor analysis [61] find a different factor structure. A study from a Persian context identified a fourth factor in addition to emotion recognition, emotion processing, and emotion expression, and reported what they called emotion tracing [64]. The Italian validation (35 items [21]) revealed two more factors in addition to the original three: Curiosity about Emotions, and Autobiographical Memory.

### The present study

The aim of this study was to validate the 60 items of the Mentalized Affectivity Scale in Hungarian, to explore its psychometric characteristics, to analyze its factor structure, and to compare the (abridged) versions adapted to other cultures with the results of the Hungarian sample.

## Materials & methods

### Research design

We collected the sample using the online LimeSurvey tool. The questionnaire, available from August 2023 to November 2023, was distributed using the snowball sampling method in various mailing lists and social networking sites. A total of 316 participants answered the questionnaire. We excluded participants who responded to less than 54 of the 60 MAS items (N = 5). The rate of missing MAS items in the reduced dataset (N = 311) was minimal (.53%). The Multiple Imputation (MI) method was used to handle missing data problems.

### Ethics statement

This study was approved by the Hungarian Ethics Committee (ETT TUKEB), reference number: BM/15404–1/2023.

Participants’ consent: The questionnaire was completed anonymously, and respondents were warned that their data would be stored electronically, which is in line with the regulations on the storage of personal data.

### Measuring instruments

Mentalized Affectivity Scale [28] – The scale contains 60 items, each rated on a 7-point Likert scale (1 – strongly disagree – 7 strongly agree). Two independent professionals translated the MAS into Hungarian, and after these versions were compared for inconsistencies, the final versions were accepted by consensus. Then a third professional translated these approved Hungarian texts back into English, again blindly and independently. The back-translations were compared with the original measures and checked for inconsistencies. The English retranslated version was verified by one of the developers of the measurement, and written permission was obtained for its use. After minor changes, the final Hungarian version was accepted by consensus of the translators.

The scale contains 15 inverted items, but on the basis of previous studies and available data files, it was difficult to identify them accurately. We examined the previously reported data on coding and then decided on the reverse items based on item-total correlations.

The original 60-item scale is divided into three subscales, with the Identifying subscale consisting of 24 items, the Processing subscale of 23, and the Expressing subscale of 13.

### Satisfaction With Life Scale (SWLS)

The Satisfaction With Life Scale [65,66] contains five statements with which the respondent is asked to rate how much he or she agrees on a 7-point scale (1 – strongly disagree, 7 – strongly agree). A higher score indicates a higher level of satisfaction with life.

The Need for Mentalizing Scale [67] contains 15 items rated on a Likert scale of 1–7 (1 – strongly disagree, 7 – strongly agree). The scale includes three factors: the Need for reading the mental states of others (7 items); the Need for untroubled interactions (5 items); and Attitude toward reading others (3 items).

The ERQ (Emotion Regulations Questionnaire) is a 10-item instrument [30,68], which consists of two subscales, Reappraisal (6 items) and Suppression (4 items) and a 7-point Likert scale from 1 (strongly disagree) to 7 (strongly agree).

The demographic question block included questions on gender, age, education, residence and marital status.

### Statistical analysis

Structural Equation Modeling was used to validate the original factor structure and the Principal Component for subsequent exploration. Model fit is acceptable if CFA if CFI > .95; RMSEA < .06 and CMIN/DF < 3 [69]. In PCA, we investigated KMO value (> 0.9 was marvellous, in the 0.80s, meritorious, in the 0.70s, middling, in the 0.60s, mediocre, in the 0.50s, miserable, and less than 0.5 would be unacceptable [70] and the Bartlett test.

We used Spearman’s correlation to measure convergent validity and the Kruskal-Wallis test for correlations with demographics.

JASP and the SPSS 29 statistical software were used for the analyses.

### Sample

The total number of respondents (descriptive statistics shown in Table 1) was 311, the youngest being 18 and the oldest 71 (mean: 34.7, st d: 13.9). Nearly 90% of the sample were women, but there were also a significant number of male respondents (37, 11.9%). Their educational attainment was higher than average, with more than half of the sample having tertiary education (58.8%) and all respondents having at least secondary education. Nearly a third of respondents lived in the capital, another half lived in rural towns and cities, but 14.5% of respondents also lived in municipalities. According to the official marital status of the sample, 38% were married, but according to their actual relationship status, more than that, 62.7%, lived in some form of relationship.

**Table 1:** Sociodemographic characteristics of the sample.

	Categories	N (%)
Age	< 30 years	137 (44.2)
	30-39 years	52 (16.7)
	40-49 years	67 (21.5)
	50-59 years	40 (12.9)
	> 60 years	14 (4.5)
	Missing	1 (0.3)
Gender	Female	272 (87.5)
	Male	37 (11.9)
	Other	1 (0.3)
	Missing	1 (0.3)
Education	Secondary	126 (40.5)
	Degree	183 (58.8)
	Missing	2 (0.6)
Habitation	Capital	117 (37.6)
	County Seat	61 (19.6)
	City	86 (27.7)
	Village	45 (14.5)
	Missing	2 (0.6)
Marital status (official)	Married	119 (38.3)
	Divorced	31 (10.0)
	Widowed	1 (0.3)
	Single	145 (46.6)
	Missing	15 (4.8)
Relationship status (valid)	In relationship	195 (62.7)
	No relationship	110 (35.4)
	Missing	6 (1.9)

## Results

### Confirmatory and exploratory factor analysis

As a first step, CFA was run on the 60 items of Greenberg [28] based on the three original factors (Identifying, Processing, and Expressing), but the model fit was unsatisfactory, CMIN/DF = 3.114; CFI = .592; RMSEA = .083). Subsequently, PCA was used to check for the existence of a fit factor structure for the 60 items. KMO value (.891) and the significant Bartlett’s test suggested that the data were suitable for factor analysis. On the basis of the ScreePlot and the Eigenvalue of the factors, three factors seemed to be the best fit, but 16 items did not perform well due to low communality (<0.25). As we had to delete a large number of items, we checked how the 35-item version, shortened by Rinaldi worked [21]. CFA did not confirm the Rinaldi factor structure either: the model fit was better than that of Greenberg, but not good (CMIN/DF = 2.829; CFI = .779; RMSEA = .077). We then ran PCA on the same 35 items to see what the factor structure was. For the five-factor solution, the KMO value (.883) and a significant Bartlett’s test suggested that the data were suitable for factor analysis. Communalities were adequate (>.35), with the five factors together explaining 54% of the variance. Only one item was included in factor 5 (MAS-07, *“I am able to wait to act on my emotions”*)*,* which could not be interpreted as a subscale. This item had a low communality in four- and three-factor solutions (.206). The resulting 34 items sorted into four factors explained 51.7%, whereas three factors 46.6% of the total variance. The items of the three-factor solution almost completely reproduced the Greenberg structure [28] in abbreviated form. Only item 35 (*“I often figure out where my emotions come from. “*) was moved from the original Identifying subscale to the Processing subscale.

### Scale statistics and internal consistency

Internal consistencies of the MAS-HU (34 items), and the three subscales were assessed using Cronbach’s alphas (Table 2). Both the 34-item scale (Cronbach’s alpha = .87) and the reliability of the subscales were good (Expressing:.83; Identifying:.86; and Processing:.82). Identifying had the highest (M = 5.71) and Expressing the lowest mean (M = 3.93).

**Table 2:** Descriptive statistics and reliability of MAS.

	Cronbach’s α	M	SD	Item-total correlations
MAS-HU (34)	.87	4.85	.95	.011−.634
Expressing (8)	.83	3.93	.66	.225−.703
Identifying (10) Processing (16)	.86 .82	5.71 4.55	.42 1,01	.271−.698 .326−.653

### Convergent validity

Next, we examined the correlation between MAS-HU and emotion-related scales. To measure general well-being, we used the single-component Satisfaction With Life Scale (SWLS), where a higher score indicates higher general well-being. The Need for Mentalizing Scale (NM) measures personal attitudes toward mentalizing and need, with three subscales, Need for reading others’ mental states (NROMS), Attitude towards reading others (ATRO) and Need for untroubled interactions (NUTI). For all three subscales, higher scores indicate higher mentalizing intention. The Emotion Regulation Questionnaire (ERQ) is divided into two subscales, with Reappraisal (R) representing the framing of difficult emotions and Suppression (S), in the opposite direction, representing the suppression of emotions (Table 3).

**Table 3:** Spearman’s correlations between MAS-HU and other scales measuring mentalization, emotion regulation and wellbeing (SWLS, NM and ERQ (N = 283-293).

	MAS-HU Processing	MAS-HU Identifying	MAS-HU Expressing
Satisfaction With Life	.195^b^	.062	.177^a^
Need for Mentalizing - Need for reading other’s mental states	.167^b^	.479^b^	.005
Need for Mentalizing - Attitude toward reading others	.023	.440^b^	−.022
Need for Mentalizing - Need for untroubled interactions	−.024	.323^b^	−.019
Emotion Regulation Questionnaire – Reappraisal	.253^b^	.252^b^	−.066
Emotion Regulation Questionnaire - Suppression	−.153^b^	−.170^b^	−.724^b^

Notes:

^a^ p < .05

^b^ p < .01

For all subscales, we observed significant correlations with the measures tested. We found most correlations for the Identifying subscale. It showed a moderately strong correlation with all MI subscales (NOMS – r_s_ = .479, p < .01; ATRO – r_s_ = .440, p < .01; NUTI – r_s _= .323; p < .01) and a significant relationship with the ERQ scales (R – r_s _= .252; p < .01; S – r_s_ = −.170; p < .01). Only weak correlations were found for the Processing subscale (max: r_s_ = .253, p < .01), while the strongest was found between Expressing and ERQ-S (r_s_ = −.724; p < .01). The direction of significant relationships was positive in all cases except for the ERQ-S scale.

### MAS-HU and socioeconomic background variables

Kruskal-Wallis tests were used to determine whether there were differences in the MAS subscale-scores between various groups of participants (Table 4).

**Table 4:** Associations between MAS-HU and sociodemographic background variables, Kruskal-Wallis H.

	MAS-HU Processing	MAS-HU Identifying	MAS-HU Expressing
Gender	.004	7.083^b^	.828
Age	15.936 ^c^	2.370	5.100
Education	9.493^b^	3.028	6.357^a^
Type of settlement	3.271	2.448	4.121
Family status	14.240^b^	4.395	11.259^b^
Partnership (yes/no)	.185	.295	22.924^c^

Notes:

^a^ p < .05

^b^ p < .01

^c^ p < .01

For the Identifying subscale, the Kruskal-Wallis test revealed significant differences only for gender (H (1) = 7.083, p = .008), with women having a higher mean ranking (Men: 118.32; Women: 159.99). The Processing subscale showed that the older the age group of the respondent, the higher the score (H (2) = 15.936, p < .001), mean ranks (under 22 years: 131). For those with higher education, the Processing (H (1) = 9.493, p = .002; Mean rank – secondary school: 136.11; higher education: 167.99), and Expressing scale (H (1) = 6.357, p = .012; Mean rank – secondary school: 139.56; higher education: 165.63) score was higher. Marital status and living in a relationship showed a similar pattern. For example, living in a relationship with a partner had a higher Expressing score (H (1) = 22.924, p < .001; Mean rank – partner: 171.16; no partner: 120.81).

## Discussion

Significant associations were found between certain sociodemographic variables and the MAS-HU subscales. In line with previous research [21,28,71] women scored higher on the Identifying subscale, which may reflect gender differences in emotional awareness and socialization processes that encourage emotional attunement among women. Age was positively associated with the Processing dimension, suggesting that emotional processing abilities may develop with age and life experience. Higher educational attainment was linked to elevated scores on both the Processing and Expressing subscales, potentially indicating that greater access to reflective and communicative practices enhances emotional self-regulation. Finally, participants living in a relationship scored significantly higher on the Expressing scale, which may be explained by the relational context offering more opportunities for emotional expression and reciprocity. These patterns are consistent with the Hungarian sample’s profile, which was predominantly female, highly educated, and largely in committed relationships, and may partially explain the elevated subscale scores observed. Compared to the Italian sample [21] which was more gender-balanced and less educated on average, our results highlight the importance of contextual and cultural factors in shaping emotional self-awareness capacities.

Emotion regulation is a very complex skill system. Its development and functioning are intertwined with the maturation of other skills of a person, as well as with the characteristics of the current relationships, environment and culture of the person. This is reflected in the difficulty of developing measures that highlight the role of mentalization skills in emotion regulation, as described in the introduction. MAS is basically concerned with the general mental regulation of emotions by the individual, but, in specific situations, it is more appropriate to use a measure that takes into account the specificities of the interpersonal context, such as the Development and validation of the Mentalizing Emotions Questionnaire (MEQ) developed by Kasper et al. [40].

An additional difficulty is the difference in the item numbers of the variants found [21,45,62] and the difference in factor structures [21, 64]. The aim of the present adaptation was to develop and validate a Hungarian-language measure that would measure mentalizing affectivity with sufficient precision (the necessary number of items), while maintaining the possibility of international comparison, and that would be compatible with the results of previous adaptations in terms of both items and factor structure.

**Fig 1:**
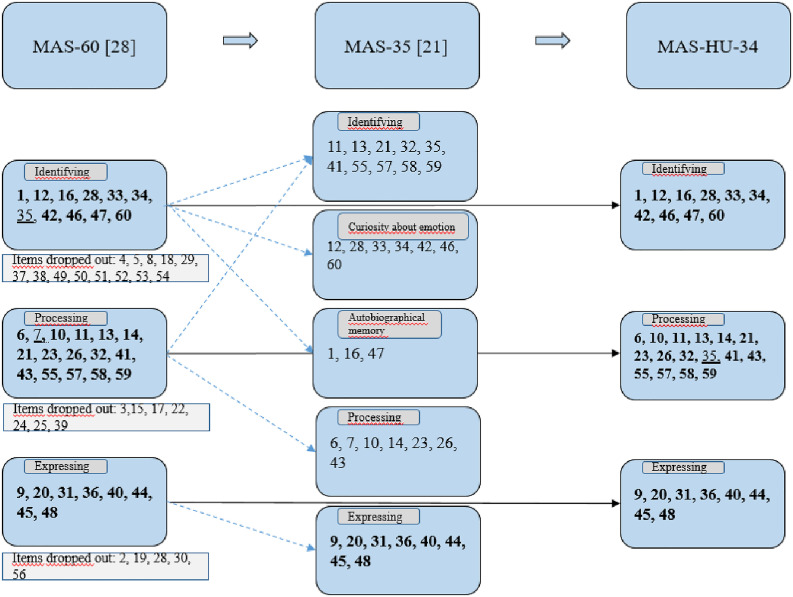
Summary of the items. Notes: During the process of the scale analyses, items shown in the square boxes were dropped out due to the results of CFA, item 35 was moved to Processing from the original Identifying subscale, item 7 was deleted due to low communality.

The confirmatory analysis did not confirm the three-factor structure of the original 60-item scale, and the exploratory analysis found that quite a few (16) items did not fit the model well. Further comparison confirmed that these items were also deleted from the final version by other studies [21]. As a next step, we therefore ran a factor structure check of Rinaldi’s 35-item corrected MAS adaptation on the Hungarian sample. The Rinaldi model basically identified the original three factors, yet they found that their model was more accurate if, in addition to the three factors (identifying, processing, and expressing), two new factors, Curiosity about emotions and Autobiographical memory, were removed. The CFA we ran did not confirm these two new factors, but with these 35 items, the original factor structure worked reasonably well. The EFA identified four factors, but one of the factors contained only one item, so it was removed. The remaining 34 items showed results nearly identical to the original factor structure developed for 60 items (Fig 1 shows the original factor structure of the 34 items, their position in the five-factor arrangement, and the results of the MAS-HU).

On the basis of the present analysis, only item 35 (“I often figure out where my emotions come from.”) was moved to a different subscale, this item being Identifying in the original scale, but in our case, it is Processing, the reason for this may be that the sentence actually contains a term referring to both (“figure out” - “where my emotions come from”), but the word order of the Hungarian and English languages may result in a different focus. While preserving this cultural specificity, but confirming the original factor structure and its content, the 34-item MAS-HU is suitable for measuring mentalizing affectivity, isolating its elements and making international comparisons.

### Suggestions and recommendations

Our findings provide valuable insights into the measurement and understanding of mentalized affectivity, contributing to both research and practical applications in the field of psychology. The validated Hungarian version of the Mentalized Affectivity Scale (MAS-HU) offers a reliable tool for assessing mentalized affectivity, enabling researchers and practitioners to examine how individuals regulate and process emotions.

Our results suggest that MAS-HU can be a valuable tool for assessing the effectiveness of programs that promote mental health by developing mentalization and emotion regulation skills. It is particularly important for the helping professionals to support burnout prevention programs in educational and workplace settings. By using MAS-HU, trainers can offer a structured approach to developing individuals’ emotion regulation skills. Training programs aimed at developing mentalization can particularly benefit from the use of MAS-HU, which can be used to examine whether the development of mentalization skills is associated with changes in the quality of an individual’s emotion regulation. This tool can also be particularly useful in the treatment of individuals with traumatic personal histories. The impact of traumatization on the development of mentalization skills is well known, and its consequences are also reflected in difficulties with emotion regulation.

Our findings demonstrate that the MAS-HU captures key components of mentalized affectivity effectively, including Identifying, Processing, and Expressing emotions. These components can be instrumental in identifying individuals’ strengths and areas for improvement in emotional regulation, facilitating tailored interventions.

Moreover, the MAS-HU’s potential for international comparisons broadens its utility, making it possible to compare findings with other adaptations of the MAS and similar constructs worldwide. Such comparisons can enrich cross-cultural research and deepen our understanding of how emotional regulation and mentalizing skills may differ across diverse populations, thereby highlighting universal versus culturally specific aspects of mentalizing affectivity.

Our results also suggest that the MAS-HU could be a valuable tool in educational and workplace settings to support emotional intelligence programs, including mentalization-focused training. By incorporating the MAS-HU into these programs, educators and employers can provide a structured approach to enhancing individuals’ emotional skills. Mentalization training, in particular, could benefit from using the MAS-HU to assess participants’ abilities to recognize, understand, and manage emotions both in themselves and others. Incorporating the MAS-HU as both an assessment and developmental tool could further personalize these programs, allowing facilitators to tailor interventions based on individual strengths and needs in mentalizing skills.

In summary, the validated MAS-HU scale provides a robust foundation for further research and practical applications in clinical, educational, and organizational contexts. Future studies can build on this work, employing the MAS-HU to explore the development and role of mentalizing affectivity across different life stages and settings.

### Limitations

One significant limitation of this study is the reliance on convenience sampling, specifically the snowball sampling method, which may introduce sampling bias. The demographics of our sample show a higher proportion of women and individuals with tertiary education, which may limit the generalizability of our findings to the broader Hungarian population. As a result, the data may not fully represent the diversity of the population, especially in terms of education, gender balance, and rural vs. urban distribution. Future research could benefit from a more diverse and representative sample to strengthen the generalizability of the findings.

Another limitation is the cross-sectional design, which captures participants’ mentalizing affectivity at a single point in time. This approach restricts our ability to examine the stability and development of mentalizing affectivity over time or in response to changing life circumstances. A longitudinal design could provide insights into the potential changes in mentalizing affectivity across different life stages or in response to specific interventions.

Lastly, although we applied the Multiple Imputation (MI) method to handle missing data, even minimal missing data can impact the statistical power and accuracy of our analyses. Future studies with larger sample sizes and potentially lower rates of missing data could enhance the robustness and reliability of results.

## Supporting information

S1 Data Data file in Excel-format. (XLSX)

S2 File MAS-HU Scale (HUN). (DOCX)

S3 File MAS-HU Scale (ENG). (DOCX)
