# Supplementary material for: Factor structure and psychometric properties of the Hungarian version of the Mentalized Affectivity Scale (MAS): A cross-sectional study
Source: PLoS One. 2025 Aug 6;20(8):e0329785. doi: 10.1371/journal.pone.0329785 (PMC12327597; doi:10.1371/journal.pone.0329785)
Supplement: S2 File — (DOCX) [file pone.0329785.s002.docx]

**Mentalizált Érzelmek Skála (MAS-HU)**

Kérem jelölje mennyire tartja a következő állításokat saját magára jellemzőnek.

1 – egyáltalán nem értek egyet ……….7 – teljes mértékben egyetértek

| 1. Gyakran foglalkoztat, hogy az érzéseim miként fakadnak korábbi élettapasztalataimból (pl. gyermekkori családi dinamikából). | 1 | 2 | 3 | 4 | 5 | 6 | 7 |
| --- | --- | --- | --- | --- | --- | --- | --- |
| 2. Képes vagyok racionálisan gondolkodni még akkor is, ha megélt érzelmeim összetettek. | 1 | 2 | 3 | 4 | 5 | 6 | 7 |
| 3. Nehézséget okoz számomra, hogy az összetett érzelmeimről beszéljek. | 1 | 2 | 3 | 4 | 5 | 6 | 7 |
| 4. Amikor eláraszt egy negatív érzés, tudom, hogyan kezeljem azt. | 1 | 2 | 3 | 4 | 5 | 6 | 7 |
| 5. Általában tisztában vagyok az érzéseim hátterében álló okokkal. | 1 | 2 | 3 | 4 | 5 | 6 | 7 |
| 6. Érzelmi élményeim megértése egy hosszú időn át zajló folyamat. | 1 | 2 | 3 | 4 | 5 | 6 | 7 |
| 7. Gyakran összezavarodom a megélt érzelmeimmel kapcsolatban. | 1 | 2 | 3 | 4 | 5 | 6 | 7 |
| 8. Képes vagyok pontosítani az érzelmeimet. | 1 | 2 | 3 | 4 | 5 | 6 | 7 |
| 9. Gyerekkori tapasztalataim ismerete hozzásegít, hogy jelenlegi érzelmeimet tágabb kontextusban szemléljem. | 1 | 2 | 3 | 4 | 5 | 6 | 7 |
| 10. Gyakran magamban tartom az érzelmeimet. | 1 | 2 | 3 | 4 | 5 | 6 | 7 |
| 11. Könnyen azonosítom magamon az „alapérzelmeket” (félelem, harag, szomorúság, öröm, meglepettség, undor). | 1 | 2 | 3 | 4 | 5 | 6 | 7 |
| 12. Jól tudom kontrollálni az érzelmeimet. | 1 | 2 | 3 | 4 | 5 | 6 | 7 |
| 13. Jól tudom kontrollálni azokat az érzelmeket, amiket nem akarok érezni. | 1 | 2 | 3 | 4 | 5 | 6 | 7 |
| 14. Segít nekem, ha átlátom az átélt érzelmeim hátterében meghúzódó okokat. | 1 | 2 | 3 | 4 | 5 | 6 | 7 |
| 15. Nem szívesen beszélem meg másokkal az érzéseimet. | 1 | 2 | 3 | 4 | 5 | 6 | 7 |
| 16. Időre van szükségem, hogy tudatosítsam, pontosan mit is érzek. | 1 | 2 | 3 | 4 | 5 | 6 | 7 |
| 17. Törekszem rá, hogy megértsem az érzelmeim összetettségét. | 1 | 2 | 3 | 4 | 5 | 6 | 7 |
| 18. Fontos számomra, hogy elismerjem saját valós érzelmeimet. | 1 | 2 | 3 | 4 | 5 | 6 | 7 |
| 19. Általában rájövök, hogy honnan erednek az érzelmeim. | 1 | 2 | 3 | 4 | 5 | 6 | 7 |
| 20. Általában inkább nem közlöm másokkal, ha érzek valamit. | 1 | 2 | 3 | 4 | 5 | 6 | 7 |
| 21. Van, amikor jobb megtartani magamnak az érzéseimet. | 1 | 2 | 3 | 4 | 5 | 6 | 7 |
| 22. Jól meg tudom különböztetni az eltérő érzelmeket, amiket megélek. | 1 | 2 | 3 | 4 | 5 | 6 | 7 |
| 23. Érzelmeim azonosítása érdeklődéssel tölt el. | 1 | 2 | 3 | 4 | 5 | 6 | 7 |
| 24. Ha egy érzés kellemetlen számomra, könnyen megszabadulok tőle. | 1 | 2 | 3 | 4 | 5 | 6 | 7 |
| 25. Gyakran tisztában vagyok az érzéseimmel, de tudatosan azt választom, hogy nem fedem fel őket a külvilág számára. | 1 | 2 | 3 | 4 | 5 | 6 | 7 |
| 26. Ha érzek valamit, az gyakran kitör belőlem. | 1 | 2 | 3 | 4 | 5 | 6 | 7 |
| 27. Erőfeszítéseket teszek az érzelmeim azonosítására. | 1 | 2 | 3 | 4 | 5 | 6 | 7 |
| 28. Pontosan meg tudom határozni azokat a gyerekkori tapasztalataimat, amelyek hatással vannak az érzéseimre és gondolkodásmódomra. | 1 | 2 | 3 | 4 | 5 | 6 | 7 |
| 29. Ha érzek valamit, azt közvetítem mások felé. | 1 | 2 | 3 | 4 | 5 | 6 | 7 |
| 30. Nem vagyok tisztában az érzelmekkel, amelyeket beszélgetés közben átélek. | 1 | 2 | 3 | 4 | 5 | 6 | 7 |
| 31. Képes vagyok gyorsan azonosítani az érzelmeimet, anélkül, hogy túl sokat gondolkodnék róluk. | 1 | 2 | 3 | 4 | 5 | 6 | 7 |
| 32. Képes vagyok megérteni az érzelmeimet a körülményeim függvényében. | 1 | 2 | 3 | 4 | 5 | 6 | 7 |
| 33. Felismerem, ha több érzelmet keveredve élek meg egy adott pillanatban. | 1 | 2 | 3 | 4 | 5 | 6 | 7 |
| 34. Érdekel, hogy többet tudjak meg arról, miért érzek bizonyos érzelmeket gyakrabban másoknál. | 1 | 2 | 3 | 4 | 5 | 6 | 7 |

Alskálák:

Azonosítás: 1, 6, 9, 14, 17, 18, 23, 27, 28, 34

Feldolgozás: 2, 4, 5, 7, 8, 11, 12, 13, 16, 19, 22, 24, 30, 31, 32, 33

Kifejezés: 3, 10, 15, 20, 21, 25, 26, 29

Fordított elemek: 3, 7, 10, 15, 16, 20, 21, 25, 30
