# Supplementary material for: Factor structure and psychometric properties of the Hungarian version of the Mentalized Affectivity Scale (MAS): A cross-sectional study
Source: PLoS One. 2025 Aug 6;20(8):e0329785. doi: 10.1371/journal.pone.0329785 (PMC12327597; doi:10.1371/journal.pone.0329785)
Supplement: S3 File — (DOCX) [file pone.0329785.s003.docx]

**Mentalized Affectivity Scale (MAS-HU)**

Please indicate the extent to which you agree or disagree with each

statement, using the scale below.

1 – Strongly disagree………7 – Strongly agree

| 1. I often think about how the emotions that I feel stem from earlier life experinces (e. g. family dinamics during childhood). | 1 | 2 | 3 | 4 | 5 | 6 | 7 |
| --- | --- | --- | --- | --- | --- | --- | --- |
| 2. I can still think rationally even if my emotions are complex. | 1 | 2 | 3 | 4 | 5 | 6 | 7 |
| 3. It is hard for me to talk about my complex emotions. | 1 | 2 | 3 | 4 | 5 | 6 | 7 |
| 4. When I am filled with a negative emotion, I know how to handle it. | 1 | 2 | 3 | 4 | 5 | 6 | 7 |
| 5. I often know the reasons why I feel the emotions. | 1 | 2 | 3 | 4 | 5 | 6 | 7 |
| 6. Understanding my emotional experince is an ongoing process. | 1 | 2 | 3 | 4 | 5 | 6 | 7 |
| 7. I am confused about the emotions that I feel. | 1 | 2 | 3 | 4 | 5 | 6 | 7 |
| 8. I am able to adjust my emotions to be more precise. | 1 | 2 | 3 | 4 | 5 | 6 | 7 |
| 9. Knowing about my childhood experinces helps to put my present emotions within larger context. | 1 | 2 | 3 | 4 | 5 | 6 | 7 |
| 10. I often keep my emotions inside. | 1 | 2 | 3 | 4 | 5 | 6 | 7 |
| 11. I can easily label "basic emotions" (fear, anger, sadness, joy and surprise) that I feel. | 1 | 2 | 3 | 4 | 5 | 6 | 7 |
| 12. I am good at controlling my emotions. | 1 | 2 | 3 | 4 | 5 | 6 | 7 |
| 13. I am good at contolling emotions that I do not want to feel. | 1 | 2 | 3 | 4 | 5 | 6 | 7 |
| 14. It helps me to know the reasons behind why I feel the way that I do. | 1 | 2 | 3 | 4 | 5 | 6 | 7 |
| 15. If I feel something, I prefer not to discuss it with others. | 1 | 2 | 3 | 4 | 5 | 6 | 7 |
| 16. It takes me a while to know how I am really feeling. | 1 | 2 | 3 | 4 | 5 | 6 | 7 |
| 17. I try to understand the complexity of my emotions. | 1 | 2 | 3 | 4 | 5 | 6 | 7 |
| 18. It is important for me to acknowledge my own true feelings. | 1 | 2 | 3 | 4 | 5 | 6 | 7 |
| 19. I often figure out where my emotions stem from. | 1 | 2 | 3 | 4 | 5 | 6 | 7 |
| 20. If I feel something, I would rather not convey it to others. | 1 | 2 | 3 | 4 | 5 | 6 | 7 |
| 21. Sometimes it is good to keep my emotions to myself. | 1 | 2 | 3 | 4 | 5 | 6 | 7 |
| 22. I am good at distinguishing between different emotions that I feel. | 1 | 2 | 3 | 4 | 5 | 6 | 7 |
| 23. I am curious about identifying my emotions. | 1 | 2 | 3 | 4 | 5 | 6 | 7 |
| 24. If a feeling makes me feel uncomfortable, I can easily get rid of it. | 1 | 2 | 3 | 4 | 5 | 6 | 7 |
| 25. I often know what I feel but choose not to reveal it outwardly. | 1 | 2 | 3 | 4 | 5 | 6 | 7 |
| 26. If I feel something, it often comes pouring out of me. | 1 | 2 | 3 | 4 | 5 | 6 | 7 |
| 27. I try to put effort into identifying my emotions. | 1 | 2 | 3 | 4 | 5 | 6 | 7 |
| 28. I can pinpoint childhood experiences that influence the way that I often think and feel. | 1 | 2 | 3 | 4 | 5 | 6 | 7 |
| 29. If I feel something, I will convey it to others. | 1 | 2 | 3 | 4 | 5 | 6 | 7 |
| 30. I am not aware of the emotions I'm feeling when in conversation. | 1 | 2 | 3 | 4 | 5 | 6 | 7 |
| 31. I can quickly identify my emotions without having to think too much about it. | 1 | 2 | 3 | 4 | 5 | 6 | 7 |
| 32. I am able to understand my emotions within the context of my surroundings. | 1 | 2 | 3 | 4 | 5 | 6 | 7 |
| 33. I can tell if I am feeling a combination os emotions at the same time. | 1 | 2 | 3 | 4 | 5 | 6 | 7 |
| 34. I am interested in learning about why I feel certain emotions more frequently than others. | 1 | 2 | 3 | 4 | 5 | 6 | 7 |

Subscales:

Identifying: 1, 6, 9, 14, 17, 18, 23, 27, 28, 34

Processing: 2, 4, 5, 7, 8, 11, 12, 13, 16, 19, 22, 24, 30, 31, 32, 33

Expressing: 3, 10, 15, 20, 21, 25, 26, 29

Reverse coded: 3, 7, 10, 15, 16, 20, 21, 25, 30
